# A Review of Embedded Artificial Intelligence Research (2023–2026): Technological Advancements, Representative Advances, and Future Prospects

**DOI:** 10.3390/mi17050586

**Published:** 2026-05-09

**Authors:** Zhaoyun Zhang

**Affiliations:** School of Electrical Engineering and Intelligentization, DongGuan University of Technology, Dongguan 523000, China; zhangzy@dgut.edu.cn

**Keywords:** embedded artificial intelligence, in-memory computing, model lightweighting, federated edge learning, edge-cloud collaboration, neuromorphic computing, embodied intelligence, full on-device training, cross-platform standardization

## Abstract

Since the publication of the “Review of Embedded Artificial Intelligence Research” in 2023, driven by innovations in hardware architectures, advances in lightweight algorithms, and the maturation of edge–cloud collaboration technologies, embedded artificial intelligence (embedded AI) has progressed from “technically feasible” to “large-scale deployment”. As a continuation of that review, this article systematically surveys the core advances in embedded AI from 2023 to 2026. At the hardware level, it examines engineering progress in non-von Neumann architectures such as compute-in-memory and neuromorphic chips, as well as heterogeneous integration technologies. At the algorithmic level, it covers dynamic adaptive lightweighting, specialized edge-side optimization of large models (including on-device large language model fine-tuning and edge diffusion models), and lightweight multimodal approaches. In terms of deployment paradigms, it discusses edge-side full training, federated edge learning, edge–cloud collaborative intelligence, and emerging paradigms. At the application level, it illustrates the “perception–decision–execution” pipeline in industrial IoT, wearable healthcare, autonomous driving, embodied intelligence, and smart agriculture. The article also analyzes core challenges including ultra-low-power design for extreme scenarios, cross-platform standardization, edge-side data security and privacy, and model robustness in complex environments. Based on these findings, four research directions are proposed to guide future work.

## 1. Introduction

Embedded artificial intelligence is a core direction of the deep integration of edge intelligence and IoT technologies. Its goal is to achieve efficient artificial intelligence inference and training on resource-constrained edge devices, aiming to overcome the inherent bottlenecks of traditional cloud-based intelligence in terms of transmission latency, bandwidth dependency, and privacy security. Previous reviews [1] have systematically summarized the foundational achievements in embedded AI, including hardware acceleration (FPGA/ASIC/GPU), model compression (quantization/pruning/binarization), and deployment modes (post-training deployment/on-device training), preliminarily establishing the technical framework of this field.

From 2023 to 2026, the development of embedded AI has been driven by multiple technological forces. At the hardware level, breakthroughs in edge-side chip manufacturing processes reaching 2 nm/3 nm nodes have significantly improved transistor integration and energy efficiency, providing a physical foundation for deploying complex AI models on power-constrained devices. Meanwhile, the increasingly prominent bottlenecks of the “memory wall” and “power wall” in traditional von Neumann architectures have spurred engineering explorations of non-von Neumann architectures such as in-memory computing and neuromorphic computing. On the algorithmic front, large model technologies are extending from the cloud to the edge, with generative AI paradigms such as Transformer, diffusion models, and lightweight large language models being implemented in embedded domains. This imposes higher demands for model lightweighting and inference efficiency, driving the evolution of lightweight strategies from static compression to dynamic adaptation and from single-modality to multimodality. At the theoretical level, the continuous advancement of low-power computing theories—particularly the maturation of methods such as subthreshold circuit design, near-threshold voltage computing, and event-driven computing—has opened new design spaces for ultralow-power embedded AI. Driven by these factors, the research focus on embedded AI is shifting from “feasible deployment” to “efficiency, intelligence, and ubiquity”.

Driven by multiple technological forces, the convergence of embedded AI with edge intelligence, IoT, and large model technologies is deepening. In terms of edge intelligence, embedded AI serves as the “edge brain” of edge nodes, undertaking core functions such as data preprocessing, real-time inference, and autonomous decision-making, forming an “end-edge-cloud” three-tier intelligent system alongside edge servers. In the IoT domain, the ubiquitous deployment of embedded AI upgrades massive IoT devices from “data collectors” to “intelligent sensing nodes”, supporting large-scale application scenarios such as industrial IoT, smart agriculture, and smart homes. Regarding large models, lightweight large models are migrating to the edge, enabling embedded devices to possess advanced intelligent capabilities such as natural language understanding, cross-modal retrieval, and generative interaction for the first time, providing critical technical support for next-generation smart terminals and embodied intelligent agents. This trend of multi-technology convergence not only expands the application boundaries of embedded AI but also endows it with deeper era significance—embedded AI is evolving from an “enabling technology” to “infrastructure for an intelligent society”.

However, alongside rapid technological advancements, embedded AI faces new challenges: ultra-low-power design in extreme scenarios (e.g., industrial high temperatures, outdoor low-power environments), cross-platform standardization compatibility for multivendor devices, security and privacy protection of edge-side multisource data, and improving model robustness in complex dynamic scenarios, all of which are critical issues constraining its large-scale deployment.

### 1.1. Review Methodology and Scope

This review follows a systematic literature search methodology, primarily conducted on the websites of IEEE, Elsevier, and MDPI. The keywords used were: (“embedded AI” OR “edge AI” OR “TinyML” OR “on-device learning” OR “on-device training”) AND (“2023” TO “2026”). The time window was set from January 2023 to December 2026. The inclusion criteria were: (i) peer-reviewed journal articles or conference papers; (ii) papers proposing novel hardware architectures, model compression techniques, deployment paradigms, or applications specifically targeting resource-constrained embedded systems.

To ensure a consistent quantitative baseline across the diverse body of literature surveyed, this review pays particular attention to standard evaluation metrics reported in the cited works. Where available, we document inference accuracy (e.g., Top-1 accuracy, F1 score, mAP), model size (number of parameters, storage footprint in MB), inference latency (milliseconds per inference) and throughput (FPS), energy efficiency (TOPS/W, μJ per inference), and compression ratio relative to the uncompressed baseline. It should be noted, however, that cross-paper comparisons of these metrics must be interpreted with caution, as the reported numbers are highly sensitive to hardware platforms (e.g., 28 nm versus 7 nm process nodes), software stacks (e.g., TensorFlow Lite versus custom runtime), and dataset characteristics. Throughout the review, we contextualize quantitative results within their original experimental settings to avoid misleading generalizations.

### 1.2. Terminology Clarification in the Embedded AI Ecosystem

The manuscript uses several related terms that are often confused. To improve conceptual clarity, Table 1 provides a clear distinction.

### 1.3. Paper Structure

As a continuation of the 2023 review [1], this paper focuses on the technological leaps from 2023 to 2026, systematically organizing core advancements and innovations in embedded artificial intelligence during this phase across hardware acceleration, model lightweighting, deployment and training paradigms, and scenario-specific applications. Building on this foundation, it delves into current challenges and prospects for future development directions. The structure of this paper is arranged as follows: Section 2 introduces next-generation hardware acceleration technologies, covering in-memory computing, neuromorphic chips, heterogeneous fusion architectures, and emerging hardware directions. Section 3 discusses advanced model lightweighting and compression strategies, including dynamic adaptive lightweighting, on-device lightweighting of large models, multimodal lightweighting, and cutting-edge strategies. Section 4 explores innovative deployment and training paradigms involving full on-device training, federated edge learning, edge-cloud collaborative intelligence, and emerging paradigms. Section 5 showcases deep applications of embedded AI in industrial IoT, wearable healthcare, smart homes, autonomous driving and smart cockpits, embodied intelligence, and smart agriculture. Section 6 analyses current core challenges. Section 7 looks ahead to future research directions. Section 8 summarizes the entire paper.

### 1.4. Incremental Content Compared with the 2023 Review

This article is a continuation of our earlier review published in 2023 [1]. The 2023 review summarized the foundational achievements of embedded AI at that time, including hardware acceleration (FPGA/ASIC/GPU), model compression (quantization, pruning, binarization), and cloud-centric deployment modes. The present review covers the period from 2023 to 2026 and extends the scope in several aspects. In terms of time frame, it focuses on the most recent three years. In hardware, the 2023 review covered traditional FPGA/ASIC/GPU accelerators and basic heterogeneous integration; the present review adds non-von Neumann architectures (compute-in-memory and neuromorphic chips) as well as 3D heterogeneous integration and chiplet technologies. In algorithms and models, the earlier work discussed lightweight CNNs (MobileNet, ShuffleNet, SqueezeNet) and standard compression methods; the present review adds dynamic adaptive lightweighting, edge-side optimisation of large language models and diffusion models, multimodal lightweighting, mixed-precision optimisation, and generative compression. In deployment paradigms, the 2023 review covered post-training deployment and limited on-device training; the present review adds full on-device training, federated edge learning, edge-cloud collaborative intelligence, model-as-a-service, and continuous learning with secure deployment. In application scenarios, in addition to general IoT and basic health monitoring, the present review also covers industrial IoT (predictive maintenance, autonomous robots), wearable healthcare (precise diagnosis, implantable devices), smart homes, autonomous driving and smart cockpits, embodied intelligence, and smart agriculture. In open challenges and future directions, the 2023 review mentioned algorithmic efficiency and basic compatibility, while the present review addresses ultra-low-power design for extreme scenarios, cross-platform standardisation and algorithm–hardware co-design, edge-side data security and privacy, and model robustness in complex dynamic environments, and proposes four research directions. The above content defines the incremental part of this review compared with the 2023 review.

## 2. Next-Generation Hardware Acceleration Technologies: Breaking Through Traditional Architectures Toward Low Power Consumption and Heterogeneous Fusion

Since 2023, research on embedded AI hardware acceleration technologies has broken through the boundaries of traditional architectures. Non-von Neumann architectures centered on Compute-in-Memory (CIM) and neuromorphic computing have become hotspots. Meanwhile, the heterogeneous integration architectures of FPGA/ASIC/GPU and the development of specialized lightweight acceleration chips have matured, forming a development pattern of “new architectures as the main focus, optimization of traditional architectures as supplementary, and heterogeneous integration as the foundation”. The core focus is on solving the long-standing “memory wall” and “power wall” issues of traditional architectures.

### 2.1. Compute-in-Memory Architecture: From Lab Validation to Engineering Implementation

Compute-in-Memory (CIM) integrates computing units and storage units deeply, performing data operations directly on the storage medium. This fundamentally eliminates frequent data transfers between storage and computing modules, significantly reducing transmission latency and power consumption. It has become a core technology direction for low-power acceleration in embedded AI. It is important to note that while the concept of CIM was proposed as early as the 20th century, its engineering implementation was long hindered by the maturity of device processes and system integration technologies. In recent years, with technological breakthroughs in novel non-volatile storage media such as resistive random-access memory (RRAM) and magnetoresistive random-access memory (MRAM), as well as the increasing maturity of 3D integration processes, CIM technology has entered a critical period for scaled development. In exploring technical pathways, researchers have formed a diversified and parallel development landscape: SRAM-based digital CIM solutions leverage reconfigurability to flexibly adapt to different models [2]; RRAM-based analogue CIM solutions demonstrate significant competitiveness in energy efficiency [3]; and 3D NAND Flash-based CIM architectures combine high storage density with parallel computing capabilities, offering high storage density advantages and providing innovative solutions for embedded edge-side high-computing demands [4].

From 2023 to 2026, the research focus of in-memory computing has evolved from material selection and device design to deep advancements in chip-level integration and adaptation for edge-side applications. Mainstream storage media include resistive random-access memory (RRAM), phase-change memory (PCM), and ferroelectric random-access memory (FeRAM). Among these, RRAM has become the preferred storage medium for embedded edge-side applications due to its high integration density, low power consumption characteristics, and good compatibility with CMOS processes, leading current engineering explorations of in-memory computing chips.

In existing research, RRAM-based in-memory computing chips have achieved edge-side inference acceleration for mainstream embedded models such as convolutional neural networks (CNNs) and lightweight transformers, with some chips achieving power consumption reductions of one to two orders of magnitude compared to traditional ASICs. For example, Wei et al. [3] proposed an RRAM-based in-memory computing macrocell employing a time-domain computation scheme. Through fully parallel matrix-vector multiplication and low-power time-to-digital converter design, it achieved a normalized energy efficiency of 1982 TOPS/W/bit, fully validating the technical potential of RRAM-based in-memory computing in ultralow-power scenarios. Simultaneously, through co-optimization of core-level parallel architecture design and sparsity-adaptive algorithms, key challenges in in-memory computing architectures—such as low computational utilization and inefficient processing of unstructured sparse data—have been effectively addressed, providing critical support for efficient model deployment at the edge.

In addition, the integration of in-memory computing with traditional hardware design has become a core path for engineering implementation. Some studies have achieved integrated optimization of “storage-computation-scheduling” by combining in-memory computing modules with FPGA or 3D integration technologies. For example, Han et al. [4] proposed a 3D-stacked in-memory computing architecture (CIMUS), which enables parallel processing of full focal plane images through block-level parallel readout design. This approach retains the low-power advantages of in-memory computing while significantly improving system processing throughput, making it suitable for edge vision scenarios such as autonomous driving, medical capsules, and augmented reality that demand high real-time performance and energy efficiency.

### 2.2. Neuromorphic Chips: From Analogue Computing to Brain-like Intelligence

Neuromorphic computing mimics the neuron and synapse structures of the human brain, adopting an event-driven computation mode that operates only when data input occurs, significantly reducing invalid power consumption. This aligns closely with the low-power requirements of embedded AI. From 2023 to 2026, research on neuromorphic chips achieved dual breakthroughs: transitioning from traditional analogue computing to “analogue-digital hybrid computing” and “event-driven deep learning”. Concurrently, studies on the compatibility of brain-like models based on spiking neural networks (SNNs) with chips have matured, showing significant progress in integration scale, energy efficiency ratio, and scenario adaptation capabilities [5].

The analogue-digital hybrid computing architecture combines the low power consumption advantages of analogue computing with the high precision benefits of digital computing. It employs analogue methods for synaptic weight storage and neuronal operations while utilizing digital approaches for data scheduling and precision calibration, effectively addressing the precision drift issues inherent in traditional analogue neuromorphic chips. For instance, Tian et al. [6] proposed a heterogeneous neuromorphic chip adopting a “sensing-storage-computing” integrated architecture. By using an analogue-digital hybrid front-end to directly convert biological signals into pulse sequences, they achieved tight coupling integration of sensors and processors, significantly reducing the overall system power consumption. Additionally, van de Burgt et al. [7] realized highly linear synaptic weight modulation based on organic electrochemical transistors, enabling high-linearity synaptic weight modulation in the analogue domain, thereby providing a new material pathway for ultralow-power analogue computing.

Research on event-driven deep learning has advanced the deep adaptation of neuromorphic chips to real-world scenarios. Leveraging the asynchronous imaging characteristics of event cameras, neuromorphic chips enable real-time processing of dynamic visual information. In applications such as autonomous driving assistance perception and industrial dynamic detection, they achieve low-latency, low-power edge-side intelligent sensing, overcoming the high bandwidth and high power consumption bottlenecks of traditional frame-based cameras. For example, Zhong et al. [5] proposed the PAICORE processor, which adopts a globally asynchronous locally synchronous (GALS) architecture. It integrates 1024 processing cores and a five-level fat quad-tree on-chip network, achieving a peak energy efficiency of 5.181 TSOPs/W in SNN inference tasks. Additionally, the event-driven data flow design significantly reduces ineffective power consumption.

The deep integration of spiking neural networks and digital circuits has become an important development direction in recent years. Traditional neuromorphic chips are mostly implemented using analogue or mixed-signal approaches, while digital implementation schemes have inherent advantages in scalability, noise immunity, and compatibility with mainstream digital systems. To the best of our knowledge, the PAICORE processor is the first to unify SNN, ANN, and on-chip Spike-timing-dependent plasticity (STDP) learning within a single architecture, achieving full paradigm support from sparse spikes to continuous data through reconfigurable computing units. It reaches 99.51% recognition accuracy on the handwritten digit recognition (N-MNIST) task and 92.01% on the DVS gesture recognition task [5]. The BioPI system proposed by Tian et al. [6] further tightly integrates event-driven analogue-to-digital converters with pipelined SNN processors, achieving 97.5% four-class classification accuracy on the MIT-BIH ECG dataset, with a single classification energy consumption of only 0.767 μJ.

The introduction of on-chip learning capabilities enables neuromorphic chips to adapt to dynamically changing edge environments. STDP-based on-chip learning mechanisms have been validated in multiple chips [5,8]. The PAICORE processor, by integrating 16 online learning cores supporting STDP, achieved incremental learning on the EMNIST dataset, improving the recognition accuracy for new categories from 4.21% to 72.67% [5]. In the field of organic neuromorphics, researchers have utilized organic electrochemical transistors to construct online-learning neuromorphic circuits, enabling reinforcement learning-based autonomous adaptation in robotic behavioral conditioning tasks [7]. These advancements indicate that neuromorphic chips are evolving from “fixed-function accelerators” to “edge intelligent agents with continuous learning capabilities”.

### 2.3. Optimization and Heterogeneous Integration of Traditional Acceleration Architectures

Traditional acceleration architectures such as FPGA/ASIC/GPU have not stagnated but are continuously optimized toward lightweight, specialized, and heterogeneous directions. Multioritecture fusion design has become a core strategy to overcome the limitations of single architectures, further consolidating their mainstream status in the field of embedded AI acceleration.

The ASIC direction focuses on the research and development of specialized lightweight AI acceleration chips. It customizes architectures for mainstream embedded lightweight models such as MobileNet, ShuffleNet, and the YOLO series. By streamlining computational units and optimizing data flow pathways, the chip area and power consumption are minimized. For example, dedicated ASIC acceleration chips for wearable devices utilize bare-die integration technology, reducing the chip area to a few square millimeters and power consumption to the microwatt level. These chips also support lightweight inference for multimodal data such as images and speech, meeting the resource-constrained requirements of consumer-grade embedded devices.

Dynamic partial reconfiguration enables FPGAs to flexibly allocate hardware resources at runtime, supporting dynamic switching between different models and tasks, making it suitable for multitasking parallel processing scenarios such as IoT edge devices [9]. Experimental results based on the Graph Convolution Network-YOLO model demonstrate that this approach achieves significant improvements in resource utilization and energy consumption [9]. Simultaneously, the integration of FPGA and MCU into System-on-Chip (SoC) has become mainstream, realizing “control-computation” integration, which greatly reduces device size and power consumption. In multitenant, multi-FPGA edge systems, the heterogeneous design management framework (HEDGY) dynamically maps different tasks to optimal FPGA design variants (such as RISC-V cores and neural network accelerators), achieving average improvements of 4.97 times in task completion time and 3.03 times in energy consumption [10]. Additionally, FPGA-based Tsetlin Machine training accelerators, serving as lightweight alternatives to DNNs, employ logic-driven on-chip learning mechanisms, achieving 6-fold and 2.54-fold improvements in energy efficiency and computational efficiency, respectively [11], providing new pathways for low-power edge-side training.

In terms of heterogeneous integration, GPU-FPGA, ASIC-in-memory computing, neuromorphic chip-FPGA, and other integrated architectures have become research hotspots. By leveraging the advantages of different architectures, they achieve collaborative optimization of “efficient computing–flexible scheduling–low-power storage”. For example, GPU-FPGA heterogeneous integration architectures are widely used in edge servers, where GPUs handle large-scale parallel computing, and FPGAs are responsible for low-latency inference and data preprocessing. The overall performance improves by over 30% compared to a single GPU, with power consumption reduced by approximately 20%. For real-time video analysis scenarios, some studies utilize dual-mirror FPGAs to achieve elastic reuse of CPU and GPU resources. Through hardware abstraction and asynchronous data transmission mechanisms, the analysis accuracy is increased to 90.35% under dynamic workloads [12], fully demonstrating the scenario adaptation advantages of heterogeneous integration.

### 2.4. Emerging Directions in Novel Hardware Acceleration Technologies

In addition to the aforementioned breakthroughs in core architectures, such as in-memory computing and neuromorphic chips, novel physics-based technologies such as photonic computing, quantum-inspired computing, system integration-oriented 3D heterogeneous integration and micronano flexible hardware technologies, have also emerged as critical extension directions in the field of embedded AI hardware acceleration from 2023 to 2026. These provide diverse pathways for extreme scenario adaptation and system performance upgrades.

#### 2.4.1. Novel Specialized Hardware Acceleration Technologies

This section focuses on hardware acceleration technologies based on novel physical mechanisms, covering photonic computing, quantum-inspired hardware, and advanced forms of in-memory analogue computing. These technologies break through the physical limitations of traditional electrical computing, endowing embedded AI with unique energy efficiency and performance advantages.

(1) Photonic Computing Chips: Utilizing optical devices such as waveguides and microring resonators to replace traditional electrical components for matrix operations, they possess inherent advantages of high bandwidth, low latency, and electromagnetic interference resistance [13]. In 2024, Hsueh et al. proposed a silicon-photonic monolithically integrated photonic-electronic linear algebra accelerator based on optical frequency combs, achieving a computational density of 2.14 TMAC/s/mm^2^ and an energy efficiency of 27.9 fJ/MAC in self-attention calculations [14]. In the same year, Perron et al. demonstrated an optical computing unit based on transient nonlinear dynamics, realizing low-power, high-robustness neuromorphic computing through soliton spectral broadening technology [15]. System-level performance modeling shows that even when accounting for opto-electronic conversion overhead, hybrid integrated optical interconnect architectures can still achieve extremely high bandwidth density and energy efficiency [16]. However, the static power consumption of photonic chips remains high, and challenges in light source integration and packaging processes remain core constraints for engineering implementation; consequently, they have not yet been commercialized on a large scale. However, the static power consumption of photonic chips remains high (typically >100 mW), and challenges in light source integration and packaging processes remain core constraints for engineering implementation; consequently, they have not yet been commercialized on a large scale.
(2) Quantum-inspired hardware: This type of hardware (such as coherent Ising machines and probabilistic bit computing) draws on the principles of quantum annealing, offering efficiency in solving combinatorial optimization problems that far surpass traditional architectures. In 2023, researchers systematically reviewed the full-stack implementation path of probabilistic bits (p-bits) from devices and architectures to algorithms. They proposed a p-bit integration scheme based on spintronic devices, leveraging their inherent stochastic switching characteristics to construct probabilistic computing hardware. This approach eliminates the need for complex pseudorandom number generators and lays a theoretical foundation for low-power AI accelerators [17]. Currently, hardware in this field primarily relies on FPGA verification, with limited general computing capabilities, and has yet to develop dedicated chips. Currently, hardware in this field primarily relies on FPGA verification, with limited general computing capabilities, and has yet to develop dedicated chips.
(3) Advanced forms of analogue in-memory computing: In-memory computing, discussed in detail in Section 2.1, has achieved independent technological advancements in the analogue domain in recent years. In 2025, Kim et al. proposed an in-memory computing architecture based on 4T1C dual-port eDRAM. By optimizing adaptive refresh and data conversion schemes, they enabled simultaneous computation and refresh operations, significantly improving the energy efficiency of analogue in-memory computing [18]. Concurrently, the International Electron Devices Meeting (IEDM) reported an 8-layer vertically stacked filament-free bulk RRAM technology, achieving 90% accuracy in wearable sensor data classification tasks. This provides a new pathway for 3D-integrated analogue in-memory computing [19]. However, this direction still faces challenges such as precision drift and device nonidealities, requiring collaborative optimization of calibration circuits and fault-tolerant algorithms. However, this direction still faces challenges such as precision drift (∼5–10% variation) and device nonidealities (e.g., read disturbance, write nonlinearity), requiring collaborative optimization of calibration circuits and fault-tolerant algorithms.

#### 2.4.2. Advanced Integration Architecture Technology

This section focuses on innovations at the system integration level, including 3D heterogeneous integration and chiplet architectures, micro/nanodevices, and flexible hardware. By optimizing integration methods and hardware forms, the integration density and scenario adaptability of embedded AI hardware are enhanced.

(1) 3D Heterogeneous Integration and Chiplet Architecture: Through vertical stacking, through-silicon vias (TSVs), and other technologies, computing chiplets, memory chiplets, and sensing chiplets are highly densely integrated, breaking through the area and interconnection bottlenecks of traditional planar integration. In 2024, Giacomini Rocha et al. conducted system-technology co-optimization (STCO) for dense edge architectures, adopting imec A10 nanosheet CMOS nodes and high-density voltage-gated spin–orbit torque MRAM (VGSOT). Combined with memory-logic fine-pitch 3D wafer-to-wafer hybrid bonding technology, experimental verification showed that 3D SRAM system integration achieved 9% power savings and 53% area reduction compared to 2D [20]. Additionally, 2.5D/3D hybrid integration technology achieved a 96 Tb/s bidirectional bandwidth between FPGA computing chiplets and photonic interface chiplets, providing high-bandwidth-density solutions for high-performance edge computing [16]. Currently, chiplet interface standards are not yet unified, and thermal management and packaging costs remain high, limiting the technology’s application to high-end embedded devices. Currently, chiplet interface standards are not yet unified, and thermal management and packaging costs remain high (estimated >$0.10 per mm^2^), limiting the technology’s application to high-end embedded devices.
(2) Micro/Nano Devices and Flexible Hardware: With the development of embedded AI in wearable, implantable, and flexible directions, novel acceleration hardware based on micro/nano devices and flexible electronics has become a research hotspot. In 2024, Shubham et al. designed flexible low-power digital circuits using amorphous silicon thin-film transistors, adopting a semilatch circuit structure to implement a 3–8 decoder on a flexible substrate, reducing the average total power consumption by 46.5% compared to traditional technologies [21] and providing core foundational circuit modules for flexible embedded intelligent systems.

### 2.5. Hardware Technology Comparison and Selection Summary

Integrating the aforementioned hardware acceleration technologies, there are significant differences in core principles, power consumption levels, computational characteristics, flexibility, maturity, and application scenarios. To facilitate researchers and engineers in selecting technologies based on application requirements, Table 2 provides a comparative summary of various hardware technologies.

From the perspective of technological evolution trends, embedded AI hardware acceleration exhibits the following characteristics:

(1) A diversified coexistence pattern. Compute-in-memory and neuromorphic chips demonstrate significant advantages in ultralow-power scenarios, but they are unlikely to completely replace the mature ecosystems of FPGA/ASIC/GPU in the short term. Over a longer period, the development landscape will feature “new architectures coexisting with traditional architectures, with heterogeneous integration as the foundation”.
(2) Scenario-driven selection. For power-sensitive applications such as wearables and implantable devices, computer-in-memory, neuromorphic chips, and analogue in-memory computing are preferred. For scenarios requiring flexible adaptation to multiple models and tasks, such as industrial control and prototype validation, FPGAs retain their advantage due to their reconfigurability. For edge servers and high-end embedded devices with high computational demands, heterogeneous fusion solutions combining GPUs and ASICs are more suitable. For scenarios with stringent size and bandwidth requirements, such as microdrones and high-end wearables, 3D heterogeneous integration and chiplet technologies are gradually becoming viable solutions.
(3) Engineering bottlenecks need to be overcome. Emerging directions such as photonic computing and quantum-inspired hardware, although demonstrating unique potential, are still far from large-scale engineering applications due to limitations in light source integration, weak generality and high packaging costs. In the future, continuous efforts are required in “algorithm–hardware” codesign, cross-platform standardization, and packaging integration processes to drive various hardware technologies from the laboratory to large-scale deployment.

### 2.6. Algorithm–Hardware Co-Design Analysis: Bridging the Gap Between Compression and Architectures

The preceding sections of Section 2 discuss hardware architectures, and Section 3 will present model compression and lightweighting algorithms. However, there is a critical missing link: how specific compression techniques map to specific hardware features to achieve actual acceleration. This section provides a co-design analysis to bridge the two domains.

In current embedded AI systems, the effectiveness of a compression technique depends heavily on whether the target hardware natively supports the resulting computational pattern. For example, unstructured pruning can achieve high sparsity ratios but delivers actual acceleration only on hardware with sparsity-aware microarchitectures, such as NVIDIA’s 2:4 structured sparsity support or custom systolic arrays equipped with zero-skipping logic. In contrast, structured pruning (e.g., channel pruning) directly reduces the dimensions of the multiply–accumulate arrays and is universally beneficial across nearly all hardware platforms.

Quantization below 8-bit requires sub-byte ALU support, which is not available on all accelerators; therefore, choosing 4-bit quantization on a device that only supports 8-bit operations yields no bandwidth or energy benefit. Knowledge distillation reduces model size without imposing special hardware requirements, making it a highly portable optimization. Neural architecture search (NAS) can be made hardware-aware by incorporating resource constraints (memory, MAC count, latency) directly into the search objective, producing models that are already adapted to the target edge device.

Dynamic adaptation techniques (e.g., multi-exit networks, conditional computation) benefit from hardware that supports early exit decisions and dynamic path selection, which FPGAs and certain NPU designs can provide. Sparse training methods, while effective for reducing update costs, require the hardware to efficiently handle sparse gradients and weight updates; neuromorphic chips with spike-timing-dependent plasticity naturally support such patterns, whereas conventional GPUs suffer from irregular memory accesses.

A key insight from recent research is that algorithm–hardware co-design is increasingly adopted as a first-class methodology. Algorithm developers should consult target hardware specifications (e.g., supported sparsity patterns, available precision modes, memory hierarchy) before selecting compression methods. Conversely, hardware designers should anticipate common compression patterns (structured sparsity, low-bit quantization, early exits) and include corresponding primitives in their microarchitectures. This bidirectional approach avoids the inefficiency of “compression-in-theory, no-benefit-in-practice” and is already evident in state-of-the-art embedded AI systems, from Google’s Edge TPU to custom CIM accelerators.

**Figure 1:**
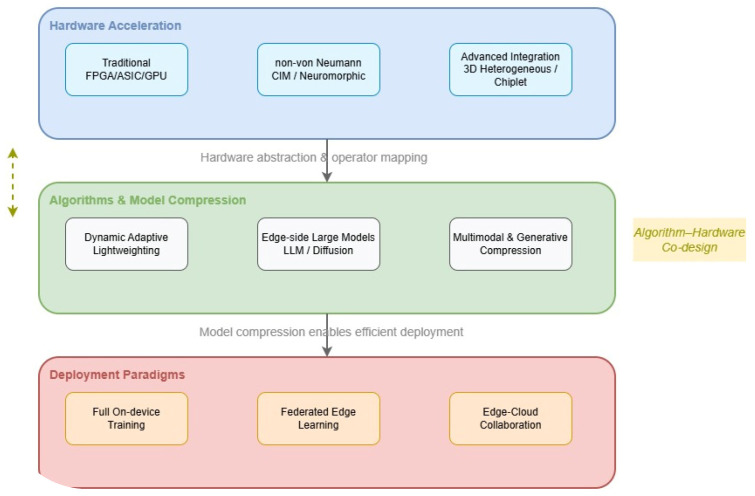
Embedded AI layered architecture.

Figure 1: Layered architecture of embedded AI technologies covered in this review. The architecture comprises three layers: hardware acceleration (bottom), algorithms and model compression (middle), and deployment paradigms (top). The “Algorithm–Hardware Co-design” component highlights the cross-layer optimization emphasized in Section 2.6. Each layer is discussed in detail in Section 2, Section 3, and Section 4, respectively.

## 3. Progressive Model Lightweighting and Compression Strategies: From Static Optimization to Dynamic Adaptation, from Single-Modality to Multi-Modality

Model lightweighting and compression are core technologies for embedded AI. From 2023 to 2026, as large models migrate to the edge and multimodal intelligence becomes a development trend, lightweighting strategies will evolve from “static single-model optimization” to “dynamic multimodel adaptation” and from “single-modality lightweighting” to “multimodality lightweighting”. Simultaneously, cross-layer collaborative optimization leveraging hardware characteristics will become a research focus, forming a lightweight design paradigm deeply integrated with “algorithm–hardware”.

### 3.1. Dynamic Adaptive Lightweighting: Real-Time Balance Between Accuracy and Resources

Traditional static lightweight methods preset fixed compression rates and network structures for models, which cannot flexibly adjust according to the real-time resource status of embedded devices (such as remaining power consumption, memory usage, and computational load) and dynamic task requirements (such as accuracy thresholds and latency constraints). This often leads to the contradiction of “resource redundancy” or “insufficient accuracy”. From 2023 to 2026, dynamic adaptive lightweighting has become the core research direction to address this issue. Its central idea is to construct multiprecision, multistructure switchable versions of the model. By real-time sensing of device resources and task requirements, it dynamically adjusts the model’s compression rate, network depth, and computational precision to achieve an optimal dynamic balance between accuracy and resources.

In existing research, the implementation paths for dynamic adaptive lightweighting mainly include dynamic network pruning, multiprecision quantization, and conditional network branching. In the area of dynamic pruning, Sharma et al. [22] proposed a CNN compression method based on dynamic parameter rank pruning. Through singular value decomposition, the convolutional kernels are subjected to low-rank decomposition. During training, the rank selection is adaptively adjusted based on data characteristics, eliminating the need for predefined pruning ratios. This approach achieved a 50% parameter compression on ResNet series models while improving classification accuracy by 2%, effectively adapting to dynamic changes in task complexity.

In the direction of multi-precision quantization, Kok et al. [23] proposed an optimization scheme integrating dynamic quantization and pruning for FPGA platforms. By adaptively adjusting quantization precision layer by layer and removing redundant network connections in real time, this method reduces the model size by 80.6% in road sign recognition tasks, with an accuracy loss of less than 2%. Moreover, the inference speed is significantly improved (the computation time of the third layer on FPGA is only 15.37% of that on a CPU), and the energy efficiency is improved by a factor of 1961.8×, making it perfectly suitable for resource-constrained embedded deployment scenarios.

Regarding conditional network branches, Selvam et al. [24] proposed the BatchCond framework, which optimizes batch processing mechanisms for conditional neural networks. Through two core steps, similarity-driven batch processing (SimBatch) and adaptive batch reorganization (ABR), inputs with similar computational patterns are grouped and processed, effectively addressing computational irregularity caused by input variations. This approach achieved up to 6.6× and an average 2.5× throughput improvement in dynamic scalable networks and early-exit networks.

Additionally, for edge-side model adaptation in dynamic environments, Kong et al. [25] designed a comprehensive video analysis system. Through the collaborative work of keyframe extractors, trigger controllers, and retraining managers, the system enables continuous responsive updates of edge models: when detecting accuracy drops exceeding adaptive thresholds, the system automatically generates optimal retraining configurations. On real datasets, this improved the detection accuracy by up to 29% while reducing the retraining time by over 50%, achieving a dynamic balance between accuracy and latency.

The aforementioned methods collectively implement the core logic of dynamic adaptive lightweighting: real-time perception of device status and task requirements, dynamically adjusting model structure and computational precision.

### 3.2. Large Model Edge Lightweighting: From Model Pruning to Distillation Acceleration

From 2023 to 2026, the deployment of large models (such as Transformer, LLaMA, and CLIP) on edge devices has become a core research direction in embedded AI. Traditional lightweighting methods struggle to adapt to the complex structures and resource requirements of large models, leading to the emergence of specialized lightweighting strategies for large models. These strategies primarily encompass model pruning, knowledge distillation, quantization-aware training, and sparsity training, forming a combined optimization paradigm of “pruning-distillation-quantization-sparsification” [26,27].

Model pruning minimizes the model size while retaining core capabilities by quantizing attention weights and neuron importance and removing redundant network layers, attention heads, and neurons. Shen et al. [28] proposed a numerical pruning method for autoregressive transformer models, which uses Newton’s method to calculate numerical importance scores for attention and MLP modules, enabling efficient pruning without retraining. Empirical results in [28] show that as the pruning ratio increases, GPU memory usage decreases and generation speed improves (see Figure 5 in [28]), while model performance is largely preserved. Li et al. [29] introduced the DP-Prune algorithm, which employs a dynamic programming-based global optimization strategy to perform structured pruning on Transformer’s attention heads and feed-forward networks. Under a 60% FLOP constraint, it achieves an 8.42% improvement in the F1 score compared to existing methods, demonstrating the benefits of global optimization for pruning effectiveness.

Knowledge distillation transfers the “knowledge” of large models to lightweight edge-side models through well-designed distillation loss functions, enabling their performance to approach that of large models. Xu et al. [30] proposed the DPKT framework, which leverages pretrained language model distillation techniques to transfer teacher model knowledge to lightweight student models. This method maintains prediction accuracy while reducing model complexity, making it suitable for edge-side resource constraints. As shown in [30], the complete DPKT achieves an accuracy of 0.8249 on the XES3G5M dataset, outperforming all baseline models, with a training time of 37.55 s per epoch.

Quantization-aware training and sparsification training embed quantization and sparsification constraints during the model training phase, enabling the model to inherently possess low-precision and high-sparsity characteristics, thereby avoiding the accuracy loss caused by postprocessing compression. Zhang et al. [31] proposed the adaptive pruning and quantization joint compression method AdaPQ, which unifies pruning importance evaluation and quantization rounding mechanisms into the same objective function. On the MobileNetV2 network, they achieved a 30% pruning rate and 8-bit quantization co-optimization, with accuracy loss controlled within 1%, inference time improved by 40%, and efficient compression without post-quantization fine-tuning, demonstrating the immense potential of combinatorial optimization.

The edge-side lightweighting of generative large models has become an emerging research hotspot during 2023–2026. Unlike traditional discriminative large models, the edge deployment of generative models, such as diffusion models and lightweight large language models, faces dual challenges. First, diffusion models require multiple iterations of denoising to generate high-quality images, leading to multiplied increases in inference latency and computational load. Second, the autoregressive inference of large language models imposes extremely high memory bandwidth requirements, where the limited storage bandwidth of edge devices easily becomes a performance bottleneck. To address these issues, current research focuses on three major technical pathways:

(1) Iteration Step Compression: Optimize sampling algorithms such as DDIM (denoising diffusion implicit model) and DPM-Solver to reduce the number of denoising steps in diffusion models, compressing inference latency from tens of seconds to seconds or even subsecond levels. Combined with knowledge distillation, multistep diffusion models are distilled into single-step or few-step generators, further reducing on-device inference overhead.
(2) Lightweight Attention Mechanisms: Replace standard self-attention in transformer-based generative models with linear attention, sparse attention, or sliding window attention, reducing computational complexity from O(n^2^) to O(n) while maintaining generation quality. The MixA framework proposed by Ahmed et al. [32] employs a hybrid attention strategy to selectively replace attention layers in pretrained ViT models. The SteLLA linear attention module, through a theoretically driven normalization mechanism, reduces inference time by 14.5% on Apple M1 and 11.0% on Raspberry Pi for the DeiT-S model (see Table 2 in [32]), while preserving classification accuracy (80.59% vs. 80.76% baseline).
(3) Distillation-Quantization Joint Optimization: Distill knowledge from large generative models into lightweight student models while integrating quantization-aware training, enabling student models to support low-bit quantization (e.g., 4-bit, 8-bit), drastically reducing storage and memory bandwidth requirements for on-device deployment.

Representative work such as the MAE microautoencoder chip proposed by Wei et al. at ISSCC 2025 [33], which adopts a 3 nm process integrating 576 MAC units, achieves 12.38 TOPS/W energy efficiency and 84.5% hardware utilization in generative AI edge vision tasks through row-based depth-first scheduling and local reuse caching mechanisms, demonstrating the feasibility of dedicated micro-NPUs for generative AI deployment on the edge.

Additionally, works such as Edge-LLaMA and TinyLLaMA enable smooth text generation of LLaMA-7B on edge devices through structured pruning and quantization-aware training, with inference latency controlled within hundreds of milliseconds.

Hardware-aware design for large edge-side models has become a research focus. By incorporating hardware computational characteristics and memory constraints during lightweight optimization, deep integration of “algorithm–hardware” is achieved, significantly improving edge-side inference efficiency. Bajwa et al. [34] systematically evaluated the effectiveness of hardware-aware pruning strategies on real-time multimedia edge devices through empirical analysis, providing important experimental foundations for hardware-aware design. For generative large models, hardware-aware design is particularly critical—for example, optimizing attention mechanisms for the matrix computation characteristics of NPUs or refining iterative sampling processes for the memory features of compute-in-memory chips can significantly enhance edge-side energy efficiency ratios.

### 3.3. Multimodal Lightweighting: Cross-Modal Feature Fusion and Resource Sharing

With the evolution of embedded AI from single-modal perception to multimodal intelligence (e.g., vision-speech-tactile fusion perception), the deployment of multimodal models on edge devices has become a critical requirement. However, multimodal models generally suffer from issues such as large parameter counts, high computational complexity, and difficulties in cross-modal feature fusion, making multimodal lightweighting a core research focus from 2023 to 2026 [35].

The core strategies for multimodal lightweighting focus on three main directions: cross-modal resource sharing, lightweight feature fusion, and modality-adaptive selection, achieving a balance between “resource efficiency” and “fusion effectiveness” through algorithmic optimization.

Cross-modal resource sharing involves designing a unified feature extraction backbone network to replace traditional independent single-modal encoders, significantly reducing redundant parameters and computational overhead. Hong et al. [35] proposed a lightweight multimodal feature isomorphic encoder (Li-MFIE), which innovatively unifies multimodal sensor data into a universal image format, eliminating the need for separate encoding modules for each modality. In human activity recognition tasks, this approach reduced the computational load (FLOPs) by 59.53% and shortened the model training time by 20%, significantly enhancing the adaptability of multimodal models for edge deployment.

Lightweight feature fusion aims to “achieve efficient fusion with low overhead”, simplifying the fusion process through techniques such as lightweight attention mechanisms and cross-modal convolutional fusion. Borsuk et al. [36] proposed a lightweight multimodal adapter that adopts the strategy of “freezing visual/text encoders + training only a lightweight projection network”. In visual object tracking tasks, this approach achieves performance comparable to full-parameter fine-tuning with only 0.461 M trainable parameters, reducing the parameter count by over 100 times, and can be directly deployed on resource-constrained edge devices such as the NVIDIA Jetson Orin NX. Tong et al. [37] introduced the FlashSloth framework, which employs an embedded visual compression design to significantly reduce the number of visual tokens while retaining visually salient information and instruction-relevant image content. This approach notably decreases training memory usage and computational complexity while maintaining high performance across various vision-language tasks.

Modal adaptive selection dynamically filters the modalities involved in computation based on the resource status of edge devices (such as remaining power and memory) and task scenarios, achieving on-demand resource allocation. Luo et al. [38] proposed the LM3D framework, which combines a depth-separable lightweight encoder with an efficient fusion module, achieving a 0.6% improvement in 3D detection accuracy on the KITTI dataset while increasing inference speed by 7.6% and reducing parameter count by 17.0%, balancing accuracy and efficiency. In the fields of industrial inspection and edge robotics, Lee et al. [39] proposed the P-LearnEdge framework, which adopts a CLIP-style dual-encoder architecture and Low-Rank Adaptation (LoRA) parameter-efficient fine-tuning. By employing 8-bit quantization, the model is compressed to 0.36 MB, achieving 2239 FPS (0.45 ms latency) for zero-shot fire detection on a Raspberry Pi 5, fully validating the feasibility of lightweight multimodal models for edge deployment.

Currently, lightweight multimodal models have been implemented in embedded scenarios such as smart speakers, wearable medical devices, and industrial robots. For example, a vision-speech multimodal model based on the fusion of MobileNet and a lightweight transformer can be deployed on smart speakers to achieve collaborative intelligence between voice wake-up and visual human recognition. The model size is less than 100 MB, with an inference latency below 200 ms, fully meeting the real-time and resource-constrained requirements of edge-side multimodal intelligence.

### 3.4. Mixed-Precision Optimization and Novel Compression Techniques: Breaking Traditional Compression Limits

By 2023, traditional model compression techniques such as quantization, pruning, and binarization had matured. From 2023 to 2026, the research focus will shift to mixed-precision optimization and novel compression technologies to overcome the accuracy and efficiency bottlenecks of traditional compression, providing efficient solutions for deploying complex models on embedded edge devices.

Mixed-precision optimization builds upon traditional quantization by finely allocating bitwidths based on the precision requirements of model layers and tasks: feature extraction layers use 4/8-bit low-precision quantization to reduce overhead, while classification and regression layers employ 16/32-bit high-precision quantization to ensure performance. Addressing the challenge of traditional layer-granularity methods in adapting to optimal bitwidths for different convolutional kernels within the same layer, Jiang et al. [40] proposed GroupQ, a group-granularity quantization method. By clustering convolutional kernel groups and employing multi-objective optimization to search for Pareto-optimal strategies, GroupQ achieved a 0.49% accuracy improvement and a 28.1% reduction in Bit Operations (BitOps) on ResNet-18 compared to HAWQ-V3. Its accompanying LMPE hardware unit further reduced power consumption by 3.6% and area by 3.9%, validating the feasibility of “algorithm–hardware” codesign.

Traditional compression relies on annotated data and suffers from difficult-to-recover precision loss. From 2023 to 2026, self-supervised compression and generative compression emerged as new directions. Self-supervised compression leverages self-supervised learning to mine redundancies, enabling compression without annotations. Huang et al. [41] addressed the sensitivity of diffusion models to timestep features by proposing the TIB maintenance strategy along with TIAR and FSC techniques. These methods enhance quantization efficiency while preserving generation quality, providing support for the deployment of diffusion models on edge devices.

Generative compression utilizes GANs (generative adversarial networks), diffusion models, etc., to restore compressed features and compensate for accuracy loss. Liu et al.’s [42] CacheQuant framework employs dynamic programming and error correction, enabling stable diffusion to achieve a 5.18× speedup and 4× compression with a CLIP score loss of only 0.02. Yang et al. [43] proposed the SDA accelerator to adapt stable diffusion models to edge FPGAs, combining W4A8 quantization (8× parameter reduction from 3.1 GB to 389 MB) with a hybrid systolic array architecture. Experimental results on the AMD-Xilinx ZCU102 FPGA show a 97.3× speedup over an ARM Cortex-A53 CPU, reducing inference time from 3.5 h to about 2.1 min, while maintaining comparable FID and CLIP scores. Ma et al.’s [44] review indicates that the combination of “distillation-pruning-quantization” can compress diffusion model denoising steps to single digits. For large language models, Anil et al. [45] proposed the LR-QAT method, freezing pretrained weights and training only LoRA and quantization parameters, outperforming traditional quantization schemes on GLUE tasks.

Currently, the integration of mixed-precision optimization and generative compression has become a research hotspot, promising to further push the limits of compression. Future efforts should focus on advancing the lightweighting of generative models themselves, reducing on-device restoration overhead, and achieving end-to-end integrated deployment of “compression-restoration”.

### 3.5. Emerging Lightweight Strategies: Extensions and Breakthroughs of Traditional Technologies

Section 3.1, Section 3.2, Section 3.3 and Section 3.4 above systematically discuss mainstream technologies such as dynamic adaptive lightweighting and large-model on-device lightweighting. From 2023 to 2026, researchers further explored several emerging strategies, which may be automated extensions of traditional methods or achieve “algorithm–hardware” collaborative innovation, completing the embedded AI lightweighting technology system.

(1) Neural architecture search (NAS): Automated network structure design. Dynamic adaptive lightweighting relies on manually designed switchable networks, while NAS enables the automated generation of on-device adaptive structures. Current research has shifted from “cloud-side search to on-device deployment” to on-device perception-driven lightweight search. Hardware-aware NAS incorporates device computing power, memory, and other resource constraints into core objectives. Garavagno et al. [46] proposed a lightweight hardware-aware NAS framework, where the search process is lightweight to be directly executable on ultralow-power microcontrollers. This framework achieves classification accuracy comparable to existing methods on multiple microvisual benchmark datasets, providing efficient automated network design solutions for extremely resource-constrained edge scenarios. Meanwhile, hypernetworks and one-shot search techniques further reduce the computational costs of the search. Currently, NAS can already generate on-device exclusive models superior to the MobileNet series on devices such as drones and smart cameras.
(2) Neural architecture reparameterization: Training-inference decoupling innovation. Traditional lightweight methods directly use compressed models for inference, while reparameterization adopts the strategy of “complex multibranch training and equivalent single-path inference”. During the training phase, multibranch structures enhance model expressiveness, and during the inference phase, they are merged into a single path, significantly reducing on-device overhead. Ding et al. [47] proposed the RepVGG method, which reparameterizes multibranch structures during training into single-layer convolutions, achieving a 2–3× inference speed improvement with no significant accuracy loss. Since 2023, this technology has been deeply integrated with quantization and pruning to form a joint optimization paradigm, which has been scaled for applications in smartphones and industrial edge devices.
(3) Hardware-Friendly Structured Sparsity: Breakthrough in Engineering Deployment. Unstructured sparsity from sparse training is difficult to translate into actual acceleration benefits. Hardware-friendly strategies adapt to hardware parallelism through structural constraints. To address the challenge of fine-grained sparse acceleration, Wu et al. [48] proposed an efficient dynamic pruning training framework for fine-grained sparse models. Combined with the proposed weight importance judgment method, sparse VGG16 and ResNet50 models can be trained from scratch, achieving a 16× compression ratio while incurring only 1/32 indexing overhead. The accompanying lightweight sparse CNN accelerator based on a modified systolic array completes the efficient deployment validation of fine-grained sparse models on edge devices. The joint optimization of sparsity and quantization can achieve dual benefits of compression and acceleration, while compute-in-memory and neuromorphic chips naturally support sparse computation, laying the foundation for sparse-aware inference engines on edge devices.
(4) Elastic Computing and Adaptive Scaling: Deepening Dynamic Lightweight. Dynamic adaptive lightweight achieves discrete adjustment by switching preset branches, while elastic computing and adaptive scaling enable continuous elastic scaling during model runtime. Li et al. [49] proposed an auction-based edge inference pricing mechanism, AERIA, which enables AI service providers to offer on-demand edge inference services based on users’ personalized requirements (accuracy, latency, budget) through dynamic pricing and resource allocation optimization. The mechanism employs multi-exit DNNs to achieve device–edge collaborative inference, significantly improving edge resource utilization and service provider revenue while meeting user quality-of-service requirements. Theoretical analysis shows that AERIA satisfies incentive compatibility and envy-freeness, ensuring fairness and trustworthiness in the edge AI market.
(5) Generative compression and diffusion model restoration: Exploration of cutting-edge technologies. From 2023 to 2026, generative compression evolves into generative AI-assisted compression, with diffusion models achieving innovative applications in precision restoration. Scribano et al. [50] proposed combining traditional image compression coding with diffusion models, conducting continuous space diffusion model training in a modelled latent space, and introducing categorized inverse process optimization to achieve efficient generative modelling of discrete latent spaces, providing new theoretical support for post-compression precision restoration. Generative knowledge distillation significantly enhances the performance of lightweight student models by generating “soft labels” and “intermediate features” through generative adversarial networks or diffusion models. Currently, generative models are mostly used for cloud or edge-assisted compression, while lightweight generative restoration on the device side remains a core research focus.

## 4. Innovative Deployment and Training Paradigms: From Cloud Dominance to End-Edge-Cloud Collaboration, from Centralized to Distributed

In 2023, the deployment and training paradigms of embedded AI were centered around cloud-based training and edge-side inference, with edge-side training limited to simple models and small-scale data, primarily constrained by the computational power and storage resources of edge devices. From 2023 to 2026, with significant advancements in edge-side hardware computing capabilities, the rapid development of lightweight training algorithms, and the increasing maturity of edge computing technologies, the deployment and training paradigms of embedded AI underwent fundamental transformations, evolving from a cloud-dominated centralized model to a distributed model of edge-cloud collaborative coordination. Full-scale edge-side training, federated edge learning, and edge-cloud collaborative training emerged as research hotspots, aiming to address core issues in traditional models such as transmission latency, bandwidth dependency, privacy security, and data silos, thereby driving embedded AI toward edge-side autonomous learning and distributed collaborative intelligence.

### 4.1. Full-Scale Edge-Side Training: From Simple Models to Complex Models for Edge-Side Autonomous Learning

Full on-device training refers to completing model training and updates directly on embedded devices without relying on cloud servers. Its core advantages are reducing data transmission costs, protecting data privacy, and enabling autonomous on-device model iteration. In 2023, on-device training could only support simple CNNs and shallow neural networks. From 2023 to 2026, with advancements in lightweight training algorithms and the computational power of on-device hardware, on-device full training has become capable of supporting complex models such as MobileNet, YOLOv8n, and small transformers. The scale of training data has also increased from thousands to tens of thousands of entries, making it suitable for embedded scenarios such as wearable devices, industrial sensors, and smart cameras.

The core technological breakthroughs in end-side full training include lightweight optimizers, incremental training, and low-precision training. In terms of lightweight optimizers, traditional optimizers occupy excessive memory due to maintaining large gradient states, making them difficult to deploy on the end side. Wu et al. [48] proposed a dynamic pruning training framework that, combined with the proposed weight importance judgment method, can obtain sparse VGG16 and ResNet50 models trained from scratch, achieving a 16× compression ratio while incurring only 1/32 indexing overhead. The accompanying lightweight sparse CNN accelerator based on a modified systolic array completes the efficient deployment validation of fine-grained sparse models on edge devices. In incremental training, continuous learning for edge environments has become a research hotspot. To address the dynamic changes in task complexity and resource status in edge environments, Li et al. [49] proposed a device–edge collaborative inference mechanism based on multi-exit DNNs. By dynamically adjusting DNN model partitioning and edge resource allocation, end devices can choose to exit at an appropriate point within the multi-exit network based on real-time inference accuracy and latency requirements. This approach significantly reduces inference latency and edge resource occupancy while meeting personalized quality-of-service requirements, thereby improving the request processing capacity of edge clusters and overall revenue. In low-precision training, the LR-QAT element adaptation method proposed by Anil et al. [45] trains only the LoRA adapter and quantization parameters while freezing pretrained weights, enabling a single training session to output multiprecision models such as BF16, INT8, and NF4, compressing end-side training memory usage to within 30% of the original FP32 model, providing a feasible path for the implementation of low-precision training on end-side devices.

Currently, full on-device training has been applied in wearable medical devices. The lightweight hardware-aware neural architecture search method proposed by Garavagno et al. [46] can automatically generate small CNNs adapted to specific hardware on ultralow-power microcontrollers and achieve personalized model updates through on-device training, maintaining accuracy comparable to cloud training in micro vision tasks such as visual wake words. Ma et al. [44] systematically reviewed efficient deployment methods of generative models on edge devices, pointing out that through the combined optimization of knowledge distillation, network pruning, and quantization-aware training, iterative denoising steps can be compressed to single digits, providing new technical pathways for full on-device training and continuous evolution of generative AI on edge devices.

### 4.2. Federated Edge Learning: Deep Integration of Distributed Training and Privacy Protection

The integration of Federated Learning (FL) and edge computing forms Federated Edge Learning (FEL), with the core idea of leveraging the distributed computing power of edge servers and embedded devices to collaboratively train models without exposing raw data. This paradigm addresses both the privacy and security issues of cloud training and reduces the computational burden on edge devices, making it a core direction for distributed training in embedded AI from 2023 to 2026.

From 2023 to 2026, the research focus of federated edge learning has deeply evolved from basic framework design to three major directions: communication efficiency optimization, heterogeneous device adaptation, and robustness enhancement. In terms of communication efficiency optimization, technologies such as gradient compression, model pruning, and sparse transmission are employed to reduce the data volume exchanged between edge devices and servers, thereby lowering latency and bandwidth consumption. Zhu et al. [51] proposed the FedLP framework, which introduces a layerwise pruning mechanism to synchronously optimize communication and computational overhead during local training and federated update phases. Theoretical analysis and experimental validation demonstrate that this method effectively alleviates system bottlenecks with negligible model performance degradation. For heterogeneous device adaptation, researchers have proposed heterogeneous federated learning algorithms to dynamically allocate training tasks and model parameters, addressing the variations in computational power and storage resources among embedded devices. Chen et al. [52] introduced the enhanced hybrid hierarchical federated edge learning (HHFEL) architecture, which adopts an online semi-asynchronous aggregation mechanism. This allows devices with stronger computational capabilities to repeatedly train local models during idle periods, effectively resolving the pain point of “low-power devices slowing down overall training progress”. Regarding robustness enhancement, techniques such as anomaly detection and Byzantine fault tolerance are utilized to address issues such as device disconnections, data contamination, and malicious attacks. Sun et al. [53] proposed the FedMFG framework, which models large-scale federated learning processes based on mean-field game theory. By calculating the cosine similarity between average field gradients and individual gradients, a reputation-aware malicious client detection and defense mechanism is constructed, improving model accuracy by 72.7% under attack scenarios.

Furthermore, the integration of federated edge learning with blockchain technology has emerged as a new research hotspot. Leveraging blockchain’s decentralized and tamper-proof characteristics enables transparent and traceable federated training processes, significantly enhancing the security and trustworthiness of model training. This approach is suitable for embedded scenarios in finance, healthcare, industry, and other fields with stringent data security requirements. Gao et al. [54] proposed a blockchain-integrated federated learning framework that employs a reputation-driven RAFT consensus protocol for lightweight blockchain coordination. Through a two-phase cross-layer optimization strategy, they jointly allocated bandwidth and computational resources, substantially improving learning accuracy, uplink throughput, and energy efficiency. Jing et al. [55] systematically reviewed the applications of federated large language models in Industry 4.0, highlighting that the deep integration of federated learning with edge computing enables cross-domain knowledge fusion and intelligent automation while protecting data privacy. This provides a comprehensive technical solution for industrial scenarios such as smart manufacturing and predictive equipment maintenance.

### 4.3. Edge–Cloud Collaborative Intelligence: Large-Scale Implementation of “Cloud–Edge–Device” Three-Tier Collaboration

Edge–cloud collaborative intelligence is the core supporting model for the large-scale implementation of embedded AI. In 2023, edge-cloud collaboration primarily involved the one-way model deployment of “cloud training-edge inference”, with edge devices acting merely as passive inference nodes, lacking bidirectional interaction capabilities with the cloud. From 2023 to 2026, edge–cloud collaborative intelligence will evolve deeply toward “bidirectional collaboration, knowledge sharing, and hierarchical intelligence”, forming a three-tier collaborative system of “cloud–edge–device”: the cloud focuses on large model training, global knowledge extraction, and cross-domain model optimization; edge servers handle regional model fusion, dynamic task scheduling, and data preprocessing; embedded devices are responsible for real-time edge inference, local data collection, and incremental training. The division of labor among these three enables efficient resource utilization and large-scale deployment of intelligent capabilities [56].

The core technologies of edge–cloud collaborative intelligence focus on three major directions: hierarchical model deployment, knowledge distillation and transfer, and dynamic task offloading, building an efficient collaborative chain:

(1) Model Hierarchical Deployment: To address the resource demands of complex models such as generative AI, Tian et al. [57] propose a bottom-up large model architecture, where edge nodes independently train small models for specific tasks, and the cloud integrates these small models into a unified multi-task large model through gating networks and linear projections, enabling cloud–edge collaborative deployment of generative AI services and effectively reducing communication overhead and end-side inference latency.
(2) Knowledge Distillation and Transfer: To solve the problem of knowledge transfer from cloud-based large models to lightweight edge-end models, Chen et al. [58] proposed a Bidirectional Distillation Hierarchical Training Framework (BDH-LM). This framework employs the streamline teacher network (STN) algorithm to gradually compress and transfer the global knowledge of cloud-based large models to edge and end-side lightweight models. Additionally, it introduces a data value-aware selection strategy, transmitting only the most informative samples, which significantly reduces communication overhead in edge-cloud collaboration while ensuring model performance.
(3) Dynamic Task Offloading: In response to the resource constraints of embedded devices and the fluctuating complexity of tasks, Alqahtani et al. [59] proposed the LITO framework, drawing inspiration from the collaborative behavior of lemur groups to model an edge-fog-cloud continuum system. Through energy-aware task allocation and collaborative scheduling strategies, the framework dynamically adjusts task deployment locations based on the remaining resources of embedded devices, the load status of edge servers, and network bandwidth. By offloading computational tasks that exceed the capabilities of the edge devices to the edge or cloud, it ultimately achieves dual optimization: reducing energy consumption by 60% and improving resource utilization by over 30%.

Currently, edge–cloud collaborative intelligence has achieved mature implementation in large-scale embedded scenarios such as intelligent transportation, industrial IoT, and smart cities. For example, Baahmed et al. [60] proposed the HiFEL-OCKT hierarchical federated edge learning method. In smart building and industrial IoT scenarios, this method addresses the collaborative adaptation issues of heterogeneous edge devices through target isomorphic clustering and multilevel knowledge transfer mechanisms, improving personalized edge knowledge capabilities by up to 87.57% and accelerating local training speed by 4.38 times. In intelligent transportation systems, onboard embedded devices perform real-time edge-side inference tasks such as vehicle detection and violation recognition. Edge servers aggregate regional traffic flow data for fusion analysis and local scheduling, while the cloud optimizes models and coordinates cross-regional traffic scheduling based on global data, forming a full-chain collaborative intelligence of “edge-side perception-edge analysis-cloud scheduling”. This significantly enhances the response speed and intelligence level of transportation systems.

### 4.4. Integrated Optimization of Training and Inference: From Separate Design to Collaborative Design

In traditional embedded AI design, model training and inference are separated: the training phase focuses on accuracy optimization without fully considering the resource constraints of edge-side inference; the inference phase only performs post-training compression and adaptation of pretrained models, which easily leads to the contradiction of “mismatch between training performance and inference requirements”. From 2023 to 2026, the integrated optimization of training and inference has become a core research direction. The central idea is to deeply incorporate the hardware characteristics, resource limitations, and accuracy requirements of edge-side inference into the model training phase, establishing a collaborative design paradigm of “training serving inference, inference guiding training”, fundamentally enhancing the deployment adaptability and inference efficiency of models on the edge side.

The implementation path for integrated training and inference optimization mainly includes hardware-aware training, inference-constrained training, and training-inference joint optimization. Hardware-aware training introduces target hardware resource constraints into the training loop, enabling the model architecture to inherently adapt to edge device characteristics. Aach et al. [61] proposed an edge AI model optimization method that constructs a closed-loop linkage mechanism between high-performance computing systems and edge devices. During training, the hardware status is fed back in real time and converted into constraints. After multiple rounds of iterative optimization, the model can efficiently adapt to target hardware without complex postprocessing, significantly reducing post-deployment performance loss and adaptation costs. Inference-constrained training embeds core inference requirement metrics during the training phase to achieve an upfront balance between accuracy and efficiency. Dong et al. [62] proposed a multi-exit DNN inference acceleration method that incorporates inference latency thresholds, confidence requirements, and edge resource allocation strategies into the training objective function. Through multidimensional joint optimization, they designed a multi-exit structure and inference path. During edge-side inference, exits can be selected as needed for early termination, minimizing end-to-end latency while ensuring that accuracy standards are met, achieving over 30% lower latency compared to traditional post-optimization methods. Training-inference joint optimization treats model training, quantization compression, and hardware mapping as unified optimization objectives, achieving full-process collaboration through closed-loop feedback. Ahmadpanah et al. [63] proposed a microservice-based collaborative deployment framework that splits large model training and edge-side hardware acceleration into coordinated microservice modules. During the training phase, parameter update strategies are dynamically adjusted based on quantization accuracy loss from the inference module. During the inference phase, hardware mapping schemes are optimized based on feature outputs from the training module. Ultimately, while maintaining core accuracy, edge-side inference efficiency is elevated to meet practical deployment standards.

### 4.5. Emerging Deployment Strategies: Driving Embedded AI Towards Dynamic, Continuous, Collaborative, and Trustworthy Evolution

Section 4.1, Section 4.2, Section 4.3 and Section 4.4 above systematically discuss mainstream deployment paradigms such as full on-device training, federated edge learning, edge-cloud collaborative intelligence, and training-inference integrated optimization. Building on this foundation, between 2023 and 2026, researchers further explored several emerging strategies, advancing embedded AI deployment from “static, fixed, isolated” to “dynamic, continuous, collaborative, and trustworthy” through four dimensions: model-as-a-service, continuous model evolution, computational architecture innovation, and secure deployment.

The model-as-a-service (MaaS) paradigm extends this concept to the edge, where embedded devices no longer rely on fixed single models. Instead, they dynamically pull lightweight models from the edge/cloud via networks, enabling dynamic sharing and on-demand allocation of model resources. Manimegala et al. [64] proposed a dynamic model adaptation framework that utilizes LoRA and convolutional bottleneck adapters. By updating only 1–5% of parameters, real-time personalized adaptation for emotion-aware speech synthesis can be achieved on edge devices such as CPUs, GPUs, and Jetson Nano, with memory overhead below 20 MB, offering an efficient technical pathway for edge-side model servitization.

Model continuous evolution is the technical deepening of full on-device training, enabling the model to continuously absorb new local data after deployment and adapt to dynamic scenarios while avoiding catastrophic forgetting. Pranesh et al. [65] propose a hybrid SRAM-RRAM hardware-aware continual learning framework for edge control applications. By employing a lightweight algorithm that buffers rapid updates in SRAM and selectively transfers stable parameters to RRAM, the framework achieves learning performance comparable to the baseline on CartPole and Pendulum tasks, while revealing that RRAM energy consumption is orders of magnitude higher than that of SRAM. This provides a hardware-level constraint awareness and design basis for lightweight continual learning strategies such as elastic weight consolidation (EWC) and memory replay.

In terms of computational architecture innovation, the serverless computing concept permeates the edge, forming lightweight implementations of function-as-a-service (FaaS). Saravana Kumar et al. [66] systematically reviewed deployment methods of serverless computing in cloud environments, analysed key performance bottlenecks such as cold start latency and runtime overhead, and proposed optimization strategies based on container prewarming and machine learning prediction of invocation timing. This lays a theoretical foundation for embedded devices to achieve a refined resource usage model of “compute on demand, pay per use”.

Secure deployment has become the core guarantee for the penetration of embedded AI into safety-critical domains, with model integrity verification receiving significant attention. Liu et al. [67] proposed a blockchain-enabled harmless model watermarking framework, replacing traditional backdoor watermarks with explainable watermarking methods. By embedding ownership information through feature impact analysis algorithms and combining blockchain for distributed storage and tamper-proof verification of watermark data, lightweight and traceable model integrity verification was achieved on the Jetson Orin Nano edge platform. Together with federated edge learning privacy protection mechanisms, this forms a “training–deployment” full lifecycle security system.

## 5. Deep Expansion of Scenario-Based Applications for Embedded AI: From Single-Point Perception to Full-Domain Intelligence

Since 2023, leveraging breakthroughs in hardware acceleration, model lightweighting, and deployment-training paradigms, embedded AI applications have extended from simple intelligent perception in single scenarios to full-process intelligence encompassing “perception-decision-execution” across industrial, medical, home, transportation, robotics, agricultural, and environmental monitoring fields. This has enabled the large-scale transition from “single-point intelligence” to “full-domain intelligence”. This section systematically outlines the application evolution, technical implementation paths, and industrial practice cases of embedded AI in six core scenarios from 2023 to 2026, demonstrating the deep integration characteristics of embedded AI across various domains.

### 5.1. Industrial Internet of Things

Industrial IoT is a key domain for embedded AI. From 2023 to 2026, embedded AI has advanced toward predictive maintenance, process optimization, and autonomous equipment control. Edge-cloud collaborative computing supports low-latency industrial applications [56]. Federated learning integrated with large language models enables cross-factory collaborative model optimization via parameter-efficient fine-tuning and communication compression [55]. Continual and reinforcement learning provide long-term adaptation under dynamic conditions [68]. Differentially private federated learning with gradient compression reduces communication and energy overhead for on-device updates while ensuring privacy [69]. Industrial predictive maintenance deploys lightweight edge models, and federated learning has shown feasibility for cross-factory modeling [55]. Embedded AI also enables autonomous control of industrial robots through on-device vision and motion planning, supported by edge-cloud architecture [56], with continual and reinforcement learning optimizing decisions under complex conditions [68]. Federated learning with differential privacy and gradient compression has been validated for industrial fault prediction [69]. Finally, low-latency, scalable collaborative systems are driving the integration of local cloud-based collaborative learning with edge-cloud intelligence [70].

### 5.2. Wearable Healthcare

Wearable healthcare demands ultra-low power, miniaturization, and privacy protection. With multimodal lightweight models and on-device full training, wearable medical devices are evolving toward precise diagnosis and personalized intervention. They collect multimodal physiological data (e.g., heart rate, blood pressure, ECG) and perform accurate on-edge diagnosis. For example, a neuromorphic system co-optimized from sensor, algorithm, and processor achieves 97.5% ECG accuracy with 0.767 μJ per classification [6]; a lightweight multimodal adapter fuses text and visual features with <0.5 M parameters [36]. Challenges include data reliability, model lightweighting, power, and privacy [71]. Embedded AI is also merging with implantable medical devices based on micro-nano components and neuromorphic chips for real-time monitoring and intervention of internal physiological signals.

### 5.3. Smart Home and Intelligent Living

Smart homes aim to break device silos through on-device intelligence and enable whole-home scenario-based interaction. With edge–cloud collaborative intelligence and multimodal lightweight models, smart home gateways deploy lightweight collaborative decision models to achieve unified scheduling and scene linkage across devices. For example, in a “coming home” scenario, the gateway uses on-device vision and speech recognition to automatically control lights, air conditioning, and curtains. This multimodal lightweight decision-making mechanism aligns with joint sensor-algorithm-processor optimization [35] and demonstrates the potential of multimodal fusion on resource-constrained devices [36]. Multimodal smart interaction integrates vision, speech, and touch for natural human–machine interaction. Challenges remain in data reliability, model lightweighting, power consumption, and privacy protection, requiring holistic consideration in system design and deployment.

### 5.4. Autonomous Driving and Smart Cockpits

Autonomous driving and intelligent cockpits demand extremely high real-time performance, reliability, and low power consumption. From 2023 to 2026, embedded AI has achieved deep integration of on-device decision-making in autonomous driving and multimodal interaction in cockpits through large-model lightweighting and edge-cloud collaboration. On-board embedded AI models, supported by edge-cloud collaborative distributed intelligence, realize adaptive cruise control, lane keeping, and automatic emergency braking with inference latency as low as tens of milliseconds [56]. In intelligent cockpits, driver status monitoring has evolved from single-vision to multimodal fusion of vision and physiological signals, achieving warning accuracy above 95% [38]. In-cabin multimodal interaction integrates voice, gesture, gaze, and facial expressions for precise “what you see is what you say” control. Additionally, child presence detection using millimeter-wave radar and vision fusion has emerged as a safety feature to monitor occupants left in rear seats after vehicle shutdown.

### 5.5. Embodied Intelligence and Intelligent Robots

Embedded AI serves as the “end-side brain” for embodied intelligence, driving robots from “cloud-controlled” to “end-side autonomous” operation. Real-time motion control and planning are core technical foundations. Neuromorphic circuits based on organic electrochemical transistors enable sensorimotor integration and reinforcement learning for maze navigation and grasping [7]. For multimodal perception, a zero-shot learning framework achieves 2239 FPS real-time inference on a low-cost edge platform [39], supporting object grasping and human–robot interaction. Hardware-software co-design is key: lightweight hardware-aware neural architecture search generates task-specific CNNs for ultra-low-power microcontrollers [46], while edge-cloud collaborative frameworks enable large-scale deployment in heterogeneous robotic systems [56]. These advances drive the closed-loop integration of “perception–decision–execution” in real-world scenarios.

### 5.6. Smart Agriculture and Environmental Monitoring

Smart agriculture and environmental monitoring demand ultra-low power, extreme environment tolerance, and wide-area connectivity. From 2023 to 2026, applications shifted from “data collection + cloud analysis” to “edge-side intelligent perception and precision decision-making”. Embedded AI chips based on lightweight vision models are integrated into drones, field stations, and irrigation equipment for real-time pest identification and precise pesticide application [38]. Smart irrigation nodes analyze soil moisture and weather at the edge, significantly reducing water usage. Wearable devices with lightweight AI monitor livestock activity and body temperature, enabling early disease detection at the edge. For environmental monitoring, low-power embedded AI nodes are deployed for water quality, air quality, and forest fire detection, where prompt-driven and zero-shot learning enable efficient fire detection without large labeled datasets [39]. Low-power wide-area networking combined with edge collaboration forms distributed monitoring networks characterized by “edge-side intelligent perception + edge collaborative decision-making” [72].

## 6. New Challenges Facing Embedded AI

From 2023 to 2026, embedded AI has achieved leapfrog development through technological breakthroughs and scenario expansion. However, as it evolves toward ultralow power consumption, ubiquity, scalability, and high security, four major new challenges—extreme scenario adaptation, cross-platform compatibility, data security and privacy, and scenario robustness—are becoming increasingly prominent, forming the core bottlenecks constraining its large-scale deployment. This section provides an in-depth analysis of these four challenges from both technical and engineering practice perspectives.

### 6.1. Ultra-Low Power Consumption and High-Reliability Design for Extreme Scenarios

The application boundaries of embedded AI are rapidly expanding from mild indoor environments to extreme scenarios such as industrial high-temperature and high-humidity conditions, outdoor frigid environments, aerospace, and implantable medical devices, imposing stringent requirements on device power consumption and reliability that surpass traditional technological limits. In terms of ultralow power consumption, self-powered sensors rely on energy harvesting technologies such as photovoltaic and thermoelectric systems, with power supply capacities of only a few tens of microwatts. Implantable medical devices need to operate stably within the human body for several years, with battery capacities constrained to extremely low levels. Traditional low-power techniques such as dynamic voltage and frequency scaling (DVFS) and clock gating can reduce dynamic power consumption. However, at advanced 3 nm/5 nm process nodes, static power consumption dominated by leakage current has become the core challenge in power control. Conventional CMOS architectures struggle to compress the overall power consumption below the microwatt level. Novel architectures such as in-memory computing and neuromorphic computing, while theoretically promising ultralow power consumption, have yet to fully overcome critical engineering hurdles. These include optimization of sleep–wake mechanisms, stability control in subthreshold operating regions, and leakage current suppression. Regarding reliability, extreme environments pose dual threats to both hardware and models: high temperatures (>85 °C) can trigger device performance drift, threshold voltage fluctuations, and interconnect electromigration, potentially leading to permanent chip damage; low temperatures (<−40 °C) can cause carrier mobility degradation and timing convergence failures; and radiation environments may induce single-event effects and total dose effects, resulting in soft errors or hard failures. Additionally, hardware nonidealities such as RRAM resistance drift and ADC nonlinearity can lead to nonlinear, unpredictable degradation in model inference accuracy, which traditional fault-tolerant algorithms struggle to comprehensively address. Achieving a dynamic balance between extreme power consumption, compression and stringent reliability constraints has emerged as a core obstacle for embedded AI in advancing into extreme scenarios.

### 6.2. Cross-Platform Standardization Compatibility and “Algorithm–Hardware” Co-Design

In the field of embedded AI, hardware devices and algorithm models exhibit significant fragmentation characteristics, severely hindering large-scale deployment and ecosystem collaborative development. At the hardware interface and instruction set level, current embedded AI hardware encompasses diverse architectures, such as FPGA, ASIC, GPU, in-memory computing, and neuromorphic chips. Even within the same category of NPU chips, ARM Ethos-U, Cadence Tensilica, and Cambricon MLU adopt entirely different programming models, memory management mechanisms, and operator libraries. Developers need to rewrite deployment code separately for each hardware type, and heterogeneous fusion chips lack unified interface standards, resulting in extremely high system integration difficulty and development costs. In terms of model format and operator compatibility, operations such as quantization, pruning, and structured sparsity generate formats such as ONNX, TFLite, and custom models, which exhibit uneven operator support across different hardware. Some hardware lacks efficient adaptation capabilities for unstructured sparsity, dynamic shapes, and custom operators, preventing theoretical compression ratios from translating into actual acceleration benefits. For example, N:M sparsity can achieve significant acceleration on GPUs but requires customized microarchitecture design to take effect on ASICs or in-memory computing chips. At the algorithm–hardware codesign level, the current approach remains predominantly separated: algorithm research focuses on accuracy and compression ratio optimization, while hardware development emphasizes energy efficiency and area control, with both lacking effective co-optimization tools and interfaces. Lightweight algorithm strategies do not fully consider hardware microarchitecture adaptability, and hardware design does not integrate model deployment requirements in advance, creating a misaligned pattern where “model design is detached from hardware, and hardware manufacturing is detached from models”, making it difficult to achieve theoretically optimal efficiency in edge-side inference. Establishing cross-platform standardized interfaces, unified operator abstraction layers, and codesign frameworks has become a critical path to resolving the fragmentation dilemma.

### 6.3. Security and Privacy Protection for Multi-Source Data on the Edge

The ubiquitous deployment of embedded AI has deeply integrated it into various fields of production and daily life, exposing it to vast amounts of sensitive data. However, the inherent limitations of edge devices in computational power, storage resources, and security protection make them “vulnerable points” for cyberattacks. At the physical security level, embedded devices are often deployed in open environments with uncontrollable physical access, allowing attackers to easily obtain hardware for side-channel attacks (extracting model parameters through power analysis or electromagnetic leakage), fault injection attacks (altering inference processes via voltage glitches or laser irradiation), or reverse engineering through physical disassembly to extract model weights from chips. Traditional encryption algorithms struggle to be efficiently deployed under the resource constraints of edge devices, requiring precise trade-offs between the attack resistance of lightweight encryption schemes and hardware overhead. In terms of data collaboration, while models such as federated learning and edge-cloud collaboration aim to avoid uploading raw data, multiple privacy leakage risks persist: gradient leakage attacks can infer training data features from shared gradients; model poisoning and backdoor attacks can implant malicious logic; and privacy leaks during the inference phase can deduce membership relationships in training data through input–output discrepancies. Regarding model supply chain security, embedded AI models undergo a multistage process of “pretraining → fine-tuning → quantization → deployment”, involving numerous development and distribution stages. Risks include model tampering, injection of malicious code, or replacement with counterfeit models. If edge devices lack effective integrity verification mechanisms, executing malicious models could lead to catastrophic consequences (e.g., autonomous vehicle malfunctions or medical device misdiagnoses). Building a comprehensive security protection system under edge resource constraints—encompassing “hardware trusted roots → secure boot → model integrity verification → runtime isolation → privacy-enhanced training”—has become a critical challenge to address.

### 6.4. Model Robustness and Generalization Capability in Complex Dynamic Scenarios

The application scenarios of embedded AI are rapidly expanding from static, controlled environments to dynamic, open, and highly interfering environments, imposing stringent requirements on model robustness and generalization capabilities that far exceed laboratory conditions. At the data distribution and environmental interference level, embedded AI models are typically trained on offline collected data. However, after deployment, factors such as lighting conditions, weather conditions, sensor noise, and user behavior patterns may undergo significant changes, leading to a cliff-like decline in model accuracy. In open-world scenarios, models often encounter new categories not present in the training set (e.g., irregular obstacles in autonomous driving or novel defects in industrial inspection), which traditional closed-set classification models struggle to handle effectively. Physical adversarial samples in natural environments (e.g., specially patterned traffic signs or artificially constructed acoustic interference) may induce models to make erroneous decisions. Regarding model generalization capabilities, existing lightweight models mostly follow a “specific dataset training → compression → deployment” pipeline, where generalization ability is constrained by the distribution assumptions of the training data. Post-deployment on edge devices, it is challenging to obtain large-scale labelled data for retraining, and traditional fine-tuning methods are prone to the risk of “catastrophic forgetting”—sacrificing original task performance while adapting to new scenarios. How to achieve continuous model self-adaptation under conditions of small samples, incremental learning, and unlabelled data has become a core challenge in enhancing generalization capabilities. In safety-critical decision reliability scenarios such as autonomous driving, medical diagnosis, and industrial control, model misjudgments can directly threaten life and property safety. However, current embedded AI models generally lack uncertainty estimation and interpretability: they cannot output prediction confidence levels, making it difficult for users to determine when to “trust the model” or “initiate human takeover”; decision processes lack traceability, complicating fault diagnosis to determine whether failures stem from sensor malfunctions, data distribution drift, or model structural flaws. Furthermore, traditional robustness enhancement techniques (e.g., adversarial training, data augmentation, and ensemble learning) often incur multiple-fold increases in computational overhead and model size expansion, creating acute conflicts with the resource constraints of embedded AI. How to enhance model robustness against distribution shifts, adversarial interference, and unknown categories without significantly increasing inference overhead has become a critical bottleneck for technological breakthroughs.

## 7. Future Research Directions

Based on the core challenges proposed in Section 6, such as ultramicro power design for extreme scenarios, cross-platform standardized compatibility, edge-side security and privacy protection, and model robustness in complex scenarios, future research in embedded artificial intelligence will focus on breakthroughs in four major directions, providing core support for the deep evolution of embedded AI into ubiquitous intelligence.

### 7.1. Ultra-Low Power Heterogeneous Fusion Architecture and Extreme Scenario Adaptation

Targeting extreme application scenarios such as industrial high temperatures, outdoor low-power environments, aerospace, and implantable medical devices, breakthroughs will be made to overcome the power consumption bottlenecks and reliability limitations of traditional hardware architectures. Key research areas include micronano computing devices and flexible embedded chips based on novel materials such as carbon nanotubes and graphene, enabling stable operation in extreme environments; deepening the integration of in-memory computing and neuromorphic computing by leveraging non-volatile memory media such as RRAM and PCM combined with event-driven brain-inspired paradigms to construct microwatt-level power, high-reliability heterogeneous fusion computing units; advancing hardware-algorithm codesigned extreme lightweight solutions through hardware-aware model structural sparsity, dynamic precision adjustment, and fault-tolerant training, achieving deep adaptation of models to ultralow power hardware while ensuring accuracy and inference efficiency in extreme scenarios.

### 7.2. Large Model Edge-Side Lightweighting and Algorithm–Hardware Co-Design

With the core objective of deploying large models on edge devices, break through the limits of traditional lightweight methods in balancing the compression ratio and accuracy. Systematically study the combined optimization paradigms of structured pruning, efficient knowledge distillation, low-precision aware training, and sparse training, forming an edge-side lightweight technology stack of “pruning-distillation-quantization-sparsification”; construct a model adaptation framework for heterogeneous hardware such as FPGA, ASIC, and in-memory computing; develop automated large-model lightweight toolchains and cross-platform deployment frameworks to lower the technical barriers for edge-side deployment; and design dedicated edge-side inference runtime systems for architectures such as Transformer and LLaMA, enhancing the execution efficiency of large models on resource-constrained devices through computational graph optimization, memory reuse, and operator fusion.

### 7.3. Edge-Cloud Full-Domain Collaborative Intelligence and Security Trust Mechanisms

To address the demand for large-scale implementation of end-edge-cloud collaboration, establish a theoretical framework for global coordination and a secure, trustworthy assurance mechanism. Collaborative optimization theory, distributed learning theory, and dynamic task scheduling mechanisms are used to build a unified “cloud–edge–end” three-tier collaborative architecture and interface standards, enabling efficient sharing of computing power, data, and knowledge. Develop lightweight encryption algorithms, device-side model watermarking, anomaly detection, and Byzantine fault tolerance technologies to adapt to the weak computational capabilities and vulnerability to attacks of end devices. Integrate differential privacy, homomorphic encryption, and federated distillation technologies to realize a distributed training paradigm where data are “usable but invisible” and models are “shareable but tamper-proof”, supporting high-security scenarios such as finance, healthcare, and industry.

### 7.4. Model Adaptability and Robustness Enhancement in Complex Dynamic Scenarios

In response to the trend of embedded AI extending into dynamic, open, and high-interference environments, the generalization and adaptability of models can be enhanced. Robustness enhancement methods such as adversarial training, domain adaptive learning, and uncertainty estimation are investigated to improve model resilience to data distribution shifts, environmental interference, and unknown inputs. Develop lightweight on-device learning algorithms such as incremental learning, few-shot learning, and meta-learning, enabling models to continuously self-iterate post-deployment using local data and adapt in real-time to scenario changes. Robustness testing benchmarks and evaluation methods for embedded AI, covering various failure modes such as adversarial attacks, environmental perturbations, and hardware drift, are established to provide reliability assurance for safety-critical applications.

## 8. Conclusions

2023–2026 is a crucial three-year period for embedded artificial intelligence to move from being technically feasible to being implemented on a large scale. Driven by multiple factors, such as hardware architecture innovation, breakthroughs in algorithm lightweighting, innovative deployment paradigms, and multidomain demand pull, embedded AI has achieved leapfrog development, completing the core transformation from “single point intelligent deployment” to “global collaborative intelligence” and building a technology system that deeply integrates the “hardware algorithm paradigm scene”.

At the hardware level, non-von Neumann architectures represented by integrated storage and computation and neural morphology chips have broken through the bottlenecks of traditional “storage walls” and “power consumption walls”, moving from laboratory verification to engineering implementation. The heterogeneous fusion and 3D heterogeneous integration of FPGA/ASIC/GPU, as well as the maturity of chip technology, have formed a diversified pattern of “coexistence of new and traditional architectures, heterogeneous fusion as the basis”, providing solid physical support for end-to-end intelligence. At the algorithmic level, breakthroughs in dynamic adaptive lightweighting, end-side specific lightweighting for large models, and multimodal lightweighting technologies have solved the deployment challenges of complex models on resource-constrained devices, especially in the exploration of end-to-end lightweighting for generative large models, enabling embedded devices to have advanced intelligent interaction capabilities for the first time. At the deployment and training paradigm level, the rise of end-to-end full training, federated edge learning, and edge cloud collaborative intelligence breaks the traditional one-way model of “cloud training end-to-end inference” and achieves the dual goals of “data privacy protection” and “distributed collaborative intelligence”. Emerging paradigms such as model servitization and end-to-end continuous learning further promote the evolution of embedded AI towards “dynamic, continuous, and trustworthy”. At the application level, embedded AI deeply penetrates six core scenarios, including industrial Internet of Things, wearable healthcare, autonomous driving, embodied intelligence, and smart agriculture, upgrading from simple perception to “perception decision execution” full process intelligence, becoming the core infrastructure for intelligent upgrades in fields such as Industry 4.0, smart cities, and mobile healthcare.

This article systematically reviews the technological progress and application practices of embedded AI from 2023 to 2026, clarifies the four core evolutionary trends of “hardware heterogeneous fusion, algorithm dynamic adaptation, paradigm distributed collaboration, and application wide intelligence”, and clarifies the internal logic and implementation path of technological development. At the same time, it is necessary to face the challenges of extreme scenario ultralow power consumption design, cross-platform standardization compatibility, end-to-end data security and privacy protection, and the robustness of complex scenario models, which are still the key bottlenecks restricting their large-scale popularization.

In the future, research on embedded AI needs to focus on “ubiquitous intelligence” as the core goal, with four major directions: first, breaking through ultralow power heterogeneous fusion architecture and extreme scenario adaptation technology, achieving a balance between “microwatt-level power consumption” and “high reliability”; second, deepening the lightweighting of the large model end side and the collaborative design of “algorithm hardware” and building an automated and cross-platform deployment toolchain; third, improving the end-to-end cloud global collaborative intelligence and securing a trustworthy mechanism, forming a security protection system with a full lifecycle of “training deployment operation”; and fourth, strengthening the adaptability and robustness of models in complex scenarios and establishing specialized evaluation benchmarks for embedded scenarios.

Embedded artificial intelligence, as the core carrier of the integration of edge intelligence and IoT technology, is evolving from “enabling technology” to “infrastructure of intelligent society”. Its continuous development will profoundly transform the way of production and life, providing efficient, safe, and inclusive intelligent solutions for industrial manufacturing, medical health, intelligent travel, ecological protection, and other fields, ultimately promoting the vision of an intelligent society with “intelligence of all things and ubiquitous perception” to become a reality.

## Figures and Tables

**Table 1:** Conceptual boundaries in the embedded AI ecosystem.

Term	Definition	Key Boundary
Embedded AI	AI executed on resource-constrained devices (MCUs, DSPs, NPUs)	Typically <1 MB RAM, <100 mW average power
Edge AI	AI performed at the network edge (gateways, edge servers)	Proximity to data source; may have more resources
On-device Intelligence	Full inference and/or training locally, no cloud fallback	Zero network dependency; closed-loop autonomy
TinyML	Machine learning on ultra-low-power devices	Power budget < 1 mW; battery life > 1 year
Edge-Cloud Collaboration	Dynamic splitting of AI tasks between edge and cloud	Hybrid model: latency/bandwidth/accuracy trade-off

**Table 2:** Comparison of Embedded AI Hardware Acceleration Technologies.

Technology Type	Core Principle	Power Consumption Level	Computational Characteristics	Flexibility	Maturity	Typical Application Scenarios	Key Citations
Compute-in-Memory (CIM)	Direct computation within storage media	Extremely low (milliwatt level)	Matrix operations are efficient, suitable for CNN/Transformer	Medium	The early stages of engineering	Wearable devices, microsensors, edge-side inference	[2,3,4]
Neuromorphic chips	Event-driven brain-inspired computing	Extremely low (submilliwatt level)	Asynchronous sparse computing, suitable for SNN	Low	Transition from laboratory to engineering	Dynamic visual processing, voice wake-up, autonomous perception	[5,6,7,8]
ASIC (dedicated chip)	Customized computing units	Low (milliwatt level)	Fixed model efficiency, concentrated computing power	Low	Mature	Consumer electronics, batch deployment in specific scenarios	[9]
FPGA	Reconfigurable logic arrays	Medium (hundreds of milliwatts level)	Parallel computing, dynamically reconfigurable	High	Mature	Industrial control, prototype verification, multitask scenarios	[9,10,11,12]
GPU	Massively Parallel Stream Processor	High (Watt-level)	General-purpose parallel computing, strong computing power	High	Mature	Edge servers, high-end embedded devices	–
Photonics Computing Chip	Optical domain matrix operations	Medium (high static power consumption)	High bandwidth, low latency	Low	Laboratory	High-speed signal processing, communication-intensive tasks	[13,14,15,16]
Quantum-Inspired Hardware	Probabilistic/Ising machine optimization	Low	Efficient combinatorial optimization	Low	Laboratory	Path planning, resource scheduling, decision optimization	[17]
Analogue In-Memory Computing	Analogue domain in-memory computing	Ultralow (submilliwatt level)	Multibit multiply accumulate, limited precision	Medium	Transition from laboratory to engineering	Ultralow power inference, micro edge devices	[18,19]
3D Heterogeneous Integration/Chiplet	Vertical stacking heterogeneous integration	Medium	High bandwidth and small area	Medium	In the early stages of engineering	High-end wearables, micro drones	[20]

**Note**: This table aims to provide a high-level qualitative comparison of hardware technologies. It should be noted that the actual performance of each hardware (e.g., TOPS/W, inference latency, energy efficiency) is highly dependent on factors such as the specific implementation process (e.g., 28 nm vs. 7 nm), model architecture (e.g., CNN vs. Transformer), data precision, sparsity, and memory hierarchy. The typical values listed in the table are only examples reported in the literature, and performance across different platforms may differ by orders of magnitude.

## Data Availability

No new data were created or analyzed in this study. Data sharing is not applicable to this article.
